# Acute aerobic exercise modulates sweetness-related food preference in individuals with nicotine dependence: the mediating role of prefrontal cortical activity

**DOI:** 10.3389/fnut.2026.1894892

**Published:** 2026-07-07

**Authors:** Hongen Liu, Yingying Zhang, Pinghan Sun, Hainan Fan, Zhao Xu

**Affiliations:** 1 Shandong Sport University, Jinan, China; 2 Guangxi Normal University, Guilin, China

**Keywords:** acute aerobic exercise, fNIRS, nicotine dependence, prefrontal cortex, sweetness-related food preference

## Abstract

**Objective:**

This study aimed to examine whether acute aerobic exercise modulates sweetness-related food preference in individuals with nicotine dependence and to further investigate the potential mediating role of prefrontal cortical activity.

**Methods:**

Sixty male university students with nicotine dependence were randomly assigned to a moderate-intensity exercise group, a high-intensity exercise group, or a rest control group, with 20 participants in each group. Participants in the exercise groups completed a 40-min aerobic cycling session, whereas those in the control group rested quietly for the same duration. Sweetness-related food preference was assessed using a 100-mm visual analogue scale at pre-intervention and immediate post-intervention. During a visual food cue paradigm, functional near-infrared spectroscopy was used to record oxygenated hemoglobin (HbO) responses in prefrontal regions of interest under high-sweetness food, low-sweetness food, and non-food control conditions.

**Results:**

A significant group × time interaction was observed for low-sweetness food preference scores (*p* = 0.008), with moderate-intensity exercise significantly increasing preference for low-sweetness foods (*p* < 0.001). For cortical responses, moderate-intensity exercise significantly enhanced right orbitofrontal cortex HbO responses under the low-sweetness food condition (*p* < 0.001). Correlation analysis showed that changes in low-sweetness food preference were positively associated with changes in right orbitofrontal cortex HbO responses in the moderate-intensity exercise group (*p* < 0.001). Exploratory mediation analysis further identified a significant indirect effect through right orbitofrontal cortex HbO responses in the association between moderate-intensity exercise and increased low-sweetness food preference (*B* = 6.56, 95% CI [4.29, 9.13]).

**Conclusion:**

Moderate-intensity acute aerobic exercise may increase preference for low-sweetness food cues in individuals with nicotine dependence, with R-OFC HbO responses showing a potential partial mediating role. These findings provide preliminary neurobehavioral evidence that acute exercise may influence low-sweetness food-cue evaluation in this population.

## 1 Introduction

Nicotine dependence (ND) is a common addictive disorder and has become a major global public health concern. Chronic nicotine exposure is associated not only with persistent responses to nicotine-related cues but also with broader alterations in cognitive control, emotion regulation, and reward processing (1). The development and maintenance of addictive behaviors may therefore involve an imbalance between the heightened motivational value of drug-related rewards and altered responsiveness to non-drug natural rewards. Examining natural reward processing may provide information that cannot be obtained solely from cigarette craving or smoking-cue reactivity, because these conventional outcomes primarily reflect drug-specific motivational processes (2, 3).

Food is a typical natural reward stimulus, and its perception and preference may be altered in individuals with ND. Food reward is multidimensional and is shaped by both the sensory properties of food and its post-ingestive nutritional consequences. Although caloric content is an important determinant of food reward, perceived sweetness is a salient sensory cue that contributes to anticipatory and hedonic food evaluation. Sweet taste and post-ingestive caloric signals therefore represent related but distinguishable dimensions of food reward (4, 5). Among food-related sensory attributes, sweet taste has strong hedonic and rewarding properties and is therefore widely regarded as an important behavioral marker of reward sensitivity (6, 7). In the present study, sweetness was selected as the dimension of interest because the visual food cue paradigm was designed to assess cue-evoked, pre-consumption reward evaluation rather than post-ingestive caloric reinforcement. Previous studies have suggested that individuals with ND may exhibit altered sweetness-related food preference, food craving, or dietary behavior (8, 9). Chronic nicotine exposure may bias reward processing toward highly salient and immediately reinforcing stimuli. Accordingly, high-sweetness food cues may retain relatively strong motivational salience, whereas low-sweetness food cues may provide a more sensitive context for detecting short-term changes in responsiveness to less salient natural rewards (7, 10, 11). This distinction provided the rationale for examining low- and high-sweetness food cues separately. Although the long-term clinical consequences of these alterations remain incompletely understood, changes in responsiveness to sweet food rewards may reflect broader adaptations within reward-related neural systems following chronic nicotine exposure. Therefore, investigating sweetness-related food preference in individuals with ND is not intended to replace the assessment of smoking-related outcomes. Rather, it provides a complementary approach for determining whether abnormalities in reward processing, and their potential modulation by exercise, extend beyond drug-specific stimuli to non-drug natural rewards.

Aerobic exercise is a safe, cost-effective, and highly feasible non-pharmacological intervention that has been widely investigated for the management of addictive behaviors and related health problems (12–14). Evidence suggests that a single bout of acute aerobic exercise can rapidly improve affective state, reduce cigarette craving and withdrawal symptoms, and enhance executive control, thereby exerting beneficial regulatory effects on ND-related behaviors (15). Compared with chronic exercise training, acute aerobic exercise is particularly suited to examining immediate exercise-induced changes in physiological and neural states, and may therefore help elucidate the rapid regulatory mechanisms through which exercise influences addiction-related behaviors (16). Importantly, the effects of acute exercise may vary with exercise intensity. Moderate-intensity exercise may support prefrontal engagement and reward-related evaluation, whereas high-intensity exercise imposes greater physiological demands and may reduce prefrontal oxygenation or cognitive efficiency (17, 18). Accordingly, the present study compared moderate- and high-intensity exercise to determine whether changes in sweetness-related food preference and PFC hemodynamic responses exhibit an intensity-dependent pattern. However, reductions in cigarette craving or smoking-cue reactivity alone cannot determine whether the effects of exercise are restricted to nicotine-related responses or reflect a broader modulation of reward processing. Examining responses to natural rewards may therefore help clarify whether acute exercise influences general reward valuation in addition to its established effects on smoking-related outcomes (19, 20).

The prefrontal cortex (PFC) is a key brain region involved in the regulation of addictive behaviors and reward-related decision-making, playing an essential role in inhibitory control, reward valuation, emotion regulation, and goal-directed behavior (21). In individuals with ND, chronic nicotine exposure may induce functional alterations in the PFC and its associated neural circuits, thereby weakening regulatory control over responses to both drug-related and natural reward stimuli (22). Meanwhile, sweet taste preference is not merely determined by gustatory perception, but is also shaped by multiple higher-order processes, including reward valuation, impulse control, and cognitive regulation (23). Specific PFC subregions, particularly the dorsolateral prefrontal cortex (DLPFC), orbitofrontal cortex (OFC), and frontal pole (FP), may contribute to the processing of hedonic experience and reward value associated with sweet stimuli (24). Previous studies have shown that acute aerobic exercise can induce changes in PFC activity and may influence behavioral choices by enhancing cognitive control and modulating reward processing (6, 25). Therefore, PFC activity may represent an important neural mechanism linking acute aerobic exercise to changes in sweetness-related food preference.

Although previous studies have investigated the effects of acute aerobic exercise on cigarette craving, withdrawal symptoms, and cognitive control from both behavioral and neurophysiological perspectives, considerably less is known about whether exercise also modulates the processing of non-drug natural rewards. This distinction is theoretically important because an effect on food-related reward would suggest that exercise-related modulation is not limited to nicotine-specific processes but may extend to broader reward-evaluation systems. Moreover, the neural mechanisms associated with exercise-induced changes in sweetness-related food preference remain unclear. Functional near-infrared spectroscopy (fNIRS) provides a feasible approach for monitoring PFC hemodynamic responses under relatively naturalistic experimental conditions, offering a useful method for elucidating the neural mechanisms through which acute exercise may influence reward-related behavior.

Accordingly, the present study used fNIRS to examine the intensity-dependent effects of acute aerobic exercise on sweetness-related food preference and PFC hemodynamic responses in individuals with ND. By using visual food cues as non-drug reward-related stimuli, we examined whether exercise-related modulation extends beyond smoking-specific outcomes to sweetness-related food-cue evaluation, hypothesizing greater effects of moderate-intensity exercise than high-intensity exercise or rest. Exploratory mediation analysis further examined whether PFC responses statistically accounted for the association between acute exercise and sweetness-related food preference.

## 2 Subjects and methods

### 2.1 Participants

#### 2.1.1 Sample size calculation

The sample size was calculated using G*Power 3.1. Based on a previous fNIRS study reporting the effect of acute aerobic exercise on prefrontal cortex activation in smokers (6), the effect size was set as Cohen’s *f* = 0.27. With *α* = 0.05 and power = 0.95 for a repeated-measures ANOVA with a within–between interaction, a minimum of 39 participants was required. Considering a potential dropout rate, 60 participants were recruited, with 20 participants in each group. The mediation analysis was conducted as an exploratory analysis and was not used as the basis for the *a priori* sample size calculation.

#### 2.1.2 Participant recruitment and eligibility criteria

Sixty male university students aged 18–25 years were recruited. Only male participants were included to reduce potential heterogeneity related to sex differences in ND, food-reward processing, and acute exercise responses. The inclusion criteria were as follows: normal BMI (18.5–22.9 kg/m^2^), ND defined as a Fagerström Test for Nicotine Dependence score ≥ 6, no history of eating disorders, psychiatric or neurological diseases, no other substance abuse, and right-handedness. Participants with taste or olfactory dysfunction, metabolic diseases, cardiovascular diseases, contraindications to exercise, or current use of medications affecting appetite, taste, mood, or central nervous system function were excluded. There were no significant differences in baseline characteristics among the three groups (Table 1).

**Table 1:** Demographic and smoking-related characteristics of participants.

Variables	Moderate (*n* = 20)	High (*n* = 20)	Rest (*n* = 20)	*f*	*p*
Age(year)	21.0 (1.5)	20.4 (1.5)	21.1 (2.4)	0.838	0.438
Height(cm)	177.8 (6.6)	178.2 (5.2)	181.7 (5.9)	2.62	0.082
Weight(kg)	69.7 (7.4)	72.4 (11.2)	73.2 (8.2)	0.816	0.447
BMI(kg/m^2^)	21.1 (1.4)	21.2 (1.5)	21.16 (1.1)	0.438	0.647
Smoking duration (years)	3.7 (1.6)	3.4 (2.0)	3.5 (1.9)	0.138	0.872
FTND (scores)	7.5 (1.1)	7.4 (1.0)	7.5 (1.1)	0.058	0.943

BMI, body mass index; FTND, Fagerström Test for Nicotine Dependence.

The study was approved by the Ethics Committee of Shandong Sport University (No. 2024006) and conducted in accordance with the Declaration of Helsinki. All participants provided written informed consent.

#### 2.1.3 Randomization and blinding

Participants were randomly assigned in a 1:1:1 ratio to the moderate-intensity exercise group, high-intensity exercise group, or rest control group, with 20 participants in each group. The random allocation sequence was generated using Microsoft Excel by an independent researcher who was not involved in participant recruitment, intervention implementation, or outcome assessment. Group allocation was concealed in sealed opaque envelopes until participants completed baseline assessments.

Due to the nature of the exercise intervention, participants and exercise supervisors could not be blinded to group allocation. However, outcome assessors responsible for administering the behavioral tests and fNIRS task were blinded to group assignment.

### 2.2 Study design

**Figure 1:**
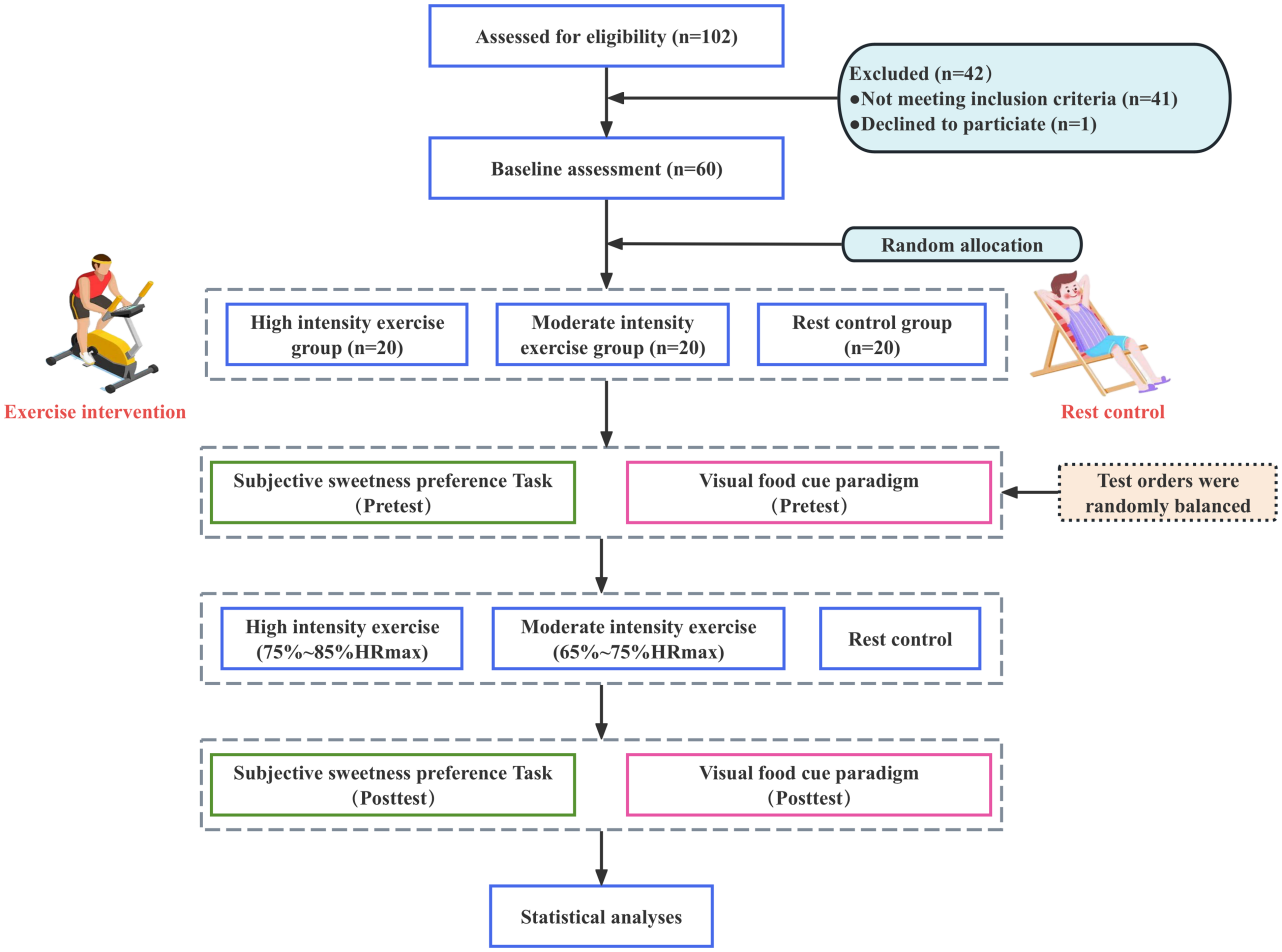
Schematic diagram of study design.

This study used a randomized controlled pretest–posttest design (Figure 1). At baseline, demographic information and questionnaire data were collected. Before and immediately after the intervention, all participants completed the visual analogue scale (VAS)-based sweetness-related food preference test and the visual food cue paradigm (VFCP). During the VFCP, fNIRS was used to measure oxygenated hemoglobin (HbO) changes in predefined PFC ROIs. The post-intervention assessment began approximately 5 min after exercise or rest cessation, and the VFCP with concurrent fNIRS recording began approximately 10 min after exercise or rest cessation. Heart rate, blood pressure, and respiration were not concurrently recorded during the post-intervention VFCP.

Before each testing session, participants were instructed to avoid strenuous exercise and alcohol consumption for 24 h, refrain from eating for 2 h, abstain from caffeinated beverages, and refrain from smoking for 2 h. The 2-h smoking abstinence period was used to minimize the acute effects of nicotine on appetite and reward sensitivity while avoiding pronounced withdrawal symptoms. All equipment was calibrated according to the manufacturer’s instructions before each session.

### 2.3 Intervention content

Participants in the exercise groups performed aerobic exercise on a cycle ergometer (Monark 928E, Sweden) according to the American College of Sports Medicine guidelines. Each exercise session lasted 40 min and consisted of a 5-min warm-up at low resistance, a 30-min main exercise phase, and a 5-min cool-down at reduced intensity. During the main exercise phase, heart rate was continuously monitored using a heart rate monitor (Polar M430, Finland) to ensure that participants remained within the prescribed intensity range. Target heart rate was maintained at 65–75% of maximum heart rate in the moderate-intensity exercise group and 75–85% of maximum heart rate in the high-intensity exercise group. Exercise intensity was adjusted by changing the cycling resistance or power output to maintain the target heart rate range. Maximum heart rate was calculated using the formula: HR_max_ = 206.9–0.67 × age (26).

The control group rested quietly for 40 min in the same quiet room. During the rest period, participants were instructed to remain seated without reading, using mobile phones, or engaging in other activities. Environmental factors, including lighting and noise, were standardized across all sessions to minimize potential confounding effects.

### 2.4 Procedure and measures

A 3 × 2 mixed design was adopted, with group as the between-subjects factor and time as the within-subjects factor. The primary behavioral outcome was the VAS score for high- and low-sweetness foods. The neural outcome was HbO change in predefined PFC ROIs during the VFCP.

#### 2.4.1 Experimental materials

Experimental stimuli were selected from 100 open-access online images, including 64 food images and 36 non-food control images matched for visual features such as color and texture. Food images were validated by 40 smokers who did not participate in the main experiment, based on the criteria proposed by Oustric et al. (27). Images were evaluated for recognition, consumption frequency, liking, and sweetness. Eligible food images had a recognition rate greater than 80%, were consumed more than twice per year, and had a liking score greater than 60 on a 100-mm VAS. According to sweetness ratings, food images were classified as high-sweetness food images (> 60) or low-sweetness food images (< 40), with perceived sweetness serving as the primary stimulus-classification dimension (Table 2).

**Table 2:** Evaluation of experimental materials.

Variables	Recognition	Frequency	Liking	Sweetness
Low-sweetness food cues	100%	4.61 ± 0.79	87.56 ± 7.29	18.33 ± 4.37
High-sweetness food cues	100%	4.92 ± 0.54	89.15 ± 7.13	79.25 ± 5.21

#### 2.4.2 Sweetness-related food preference test

**Figure 2:**
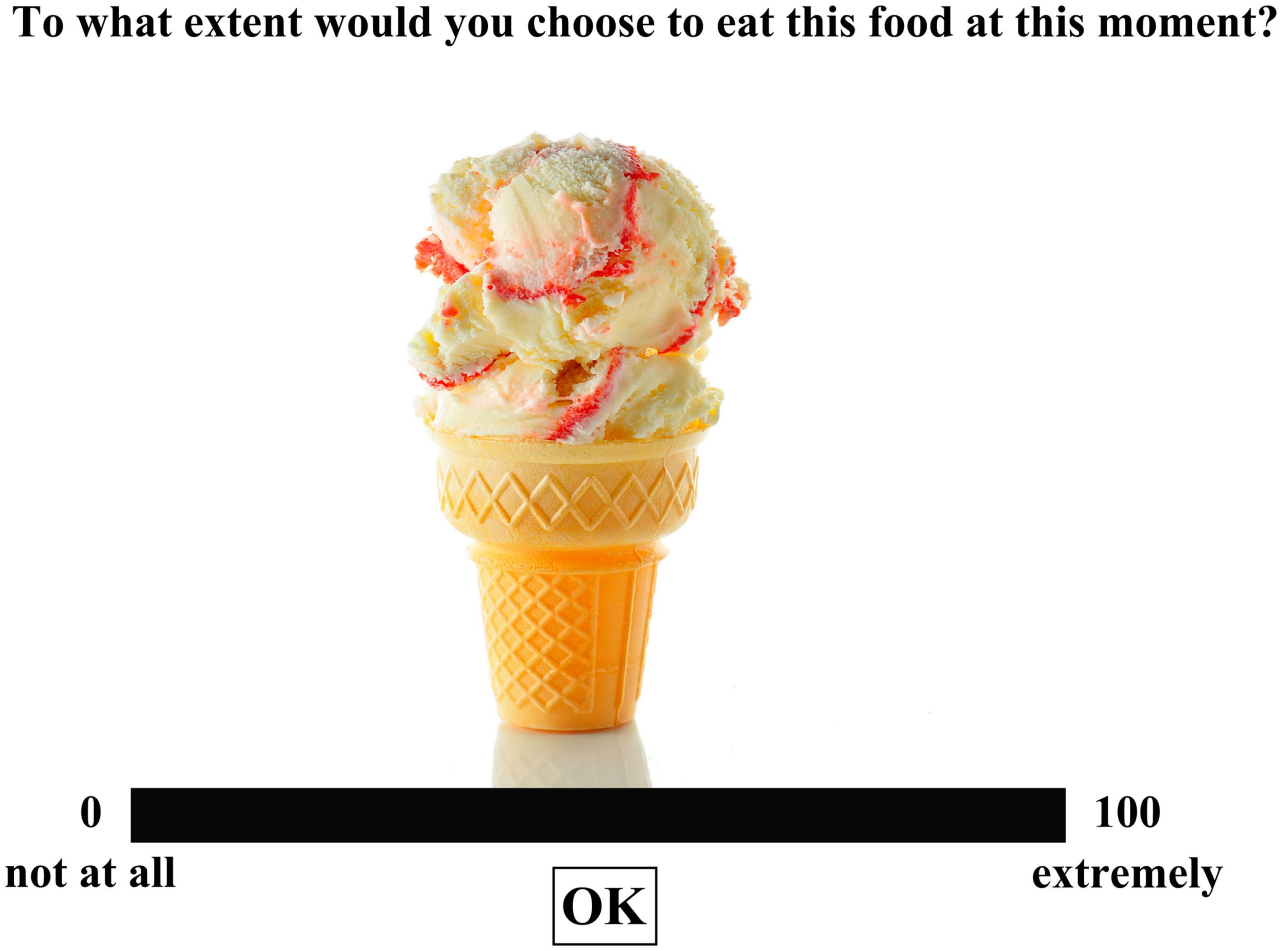
VAS-based sweetness-related food preference test.

Sweetness-related food preference was assessed using visual food images and a 100-mm VAS. In each test, 16 food images were presented one at a time in a randomized order, including 8 high-sweetness food images and 8 low-sweetness food images. For each image, participants were asked to answer the question: “To what extent would you choose to eat this food at this moment?” The VAS was displayed on the screen and anchored from “not at all” to “extremely.” Participants indicated their preference by selecting a position along the scale. The score ranged from 0 to 100, with higher scores indicating stronger preference. After each response was recorded, the next image was automatically presented. Mean VAS preference scores were calculated separately for high- and low-sweetness food cues for subsequent analysis (Figure 2).

#### 2.4.3 Visual food cue paradigm

The VFCP was adapted from the paradigm of Killgore et al. (28). Color images were presented against a white background on a computer screen positioned 30 cm in front of the participants. The stimuli included high-sweetness food images, such as cotton candy, doughnuts, and lollipops; low-sweetness food images, such as tomatoes, whole wheat bread, and milk; and non-food control images matched for visual features such as color and texture. Participants were instructed to observe each image carefully; for food images, they were asked to imagine the taste of the depicted food. During the VFCP, fNIRS was used to record HbO changes in predefined PFC ROIs. Participants were instructed to maintain attention to the presented stimuli throughout the task. No overt behavioral responses or eye-tracking measures were recorded during the paradigm.

**Figure 3:**
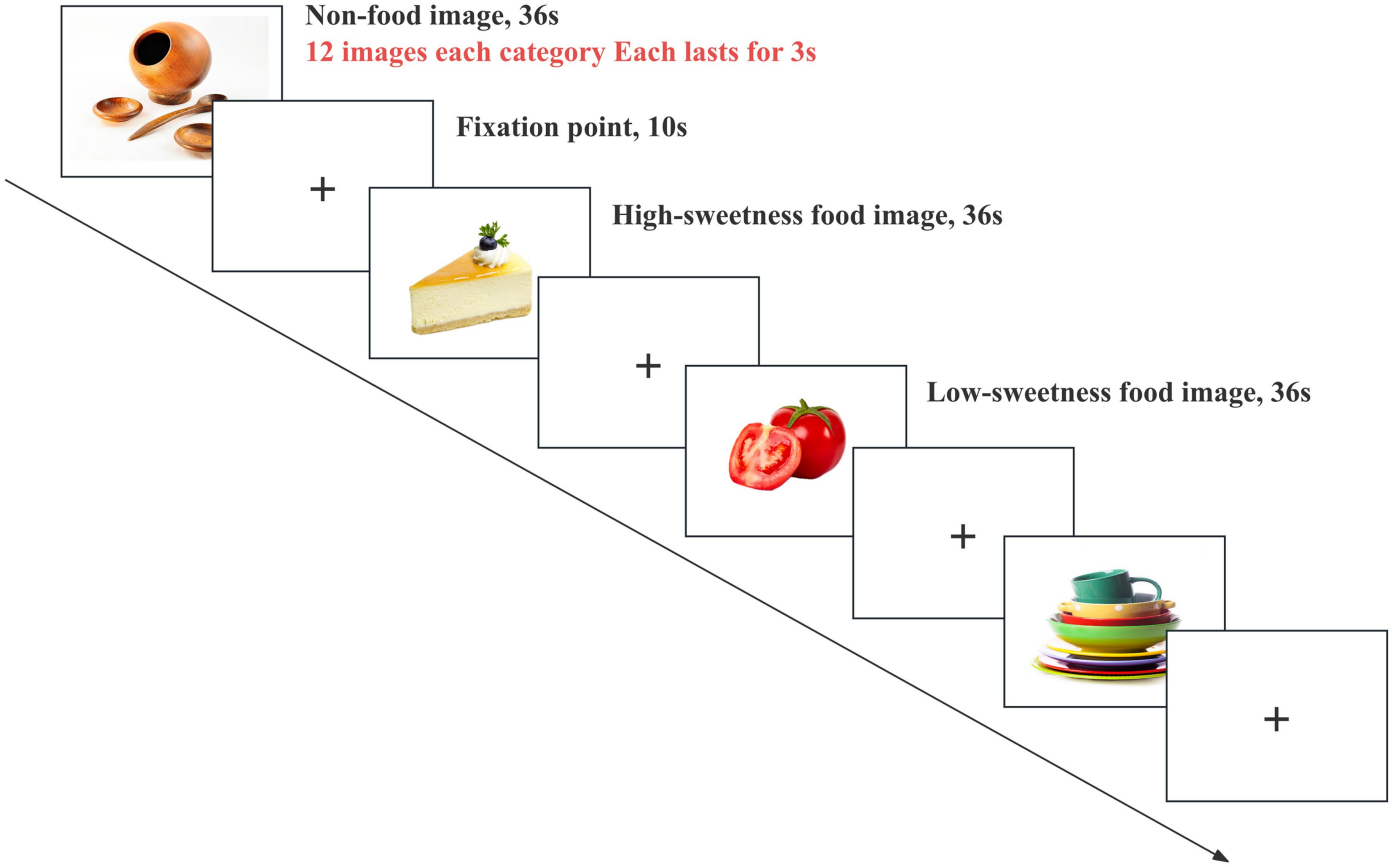
Visual food cue fNIRS task.

The VFCP consisted of 6 blocks, with each block containing 12 images from the same condition, resulting in 72 images in total. These images were different from those used in the VAS test. Each image was presented once for 3 s, and the order of blocks and images within each block was randomized using E-Prime. A 10-s fixation cross was displayed between blocks. The total duration of the paradigm was 266 s and was kept constant across all participants (Figure 3).

#### 2.4.4 fNIRS data acquisition

**Figure 4:**
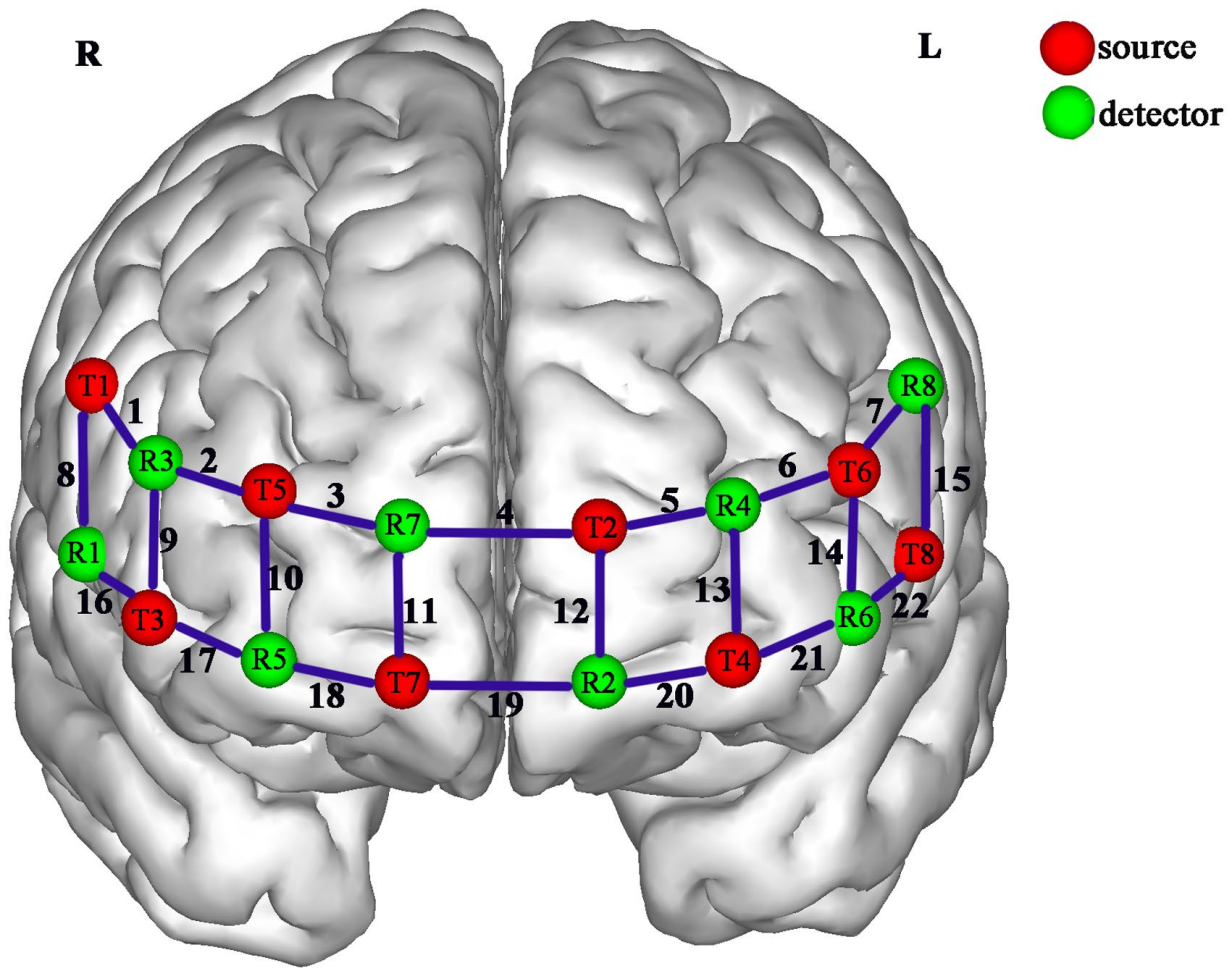
PFC channel layout (front view).

Hemodynamic responses in the PFC were recorded during the VFCP using an fNIRS system (Shimadzu, Japan) with three wavelengths of near-infrared light: 780, 805, and 830 nm. The sampling rate was 13.33 Hz. The optode cap was positioned according to the international 10–20 system. A multichannel probe set consisting of eight emitters and eight detectors was used, forming 22 measurement channels, with a source–detector distance of 3 cm (Figure 4). No short-separation channels were included in the probe configuration; therefore, short-channel regression was not performed. Channel coordinates were digitized using a 3D digitizer (Polhemus, USA), registered to standard brain space using SPM, and assigned to predefined PFC ROIs according to the Brodmann atlas (Table 3).

**Table 3:** Channels corresponding to PFC ROI.

PFC ROI	Right area channels	Left area channels
Dorsolateral prefrontal cortex	1	7
Ventrolateral prefrontal cortex	8, 16	15, 22
Frontal polar cortex	2, 3, 4, 9, 10, 11	5, 6, 12, 13, 14
Orbitofrontal cortex	17, 18, 19	20, 21

### 2.5 Data processing and statistical analysis

#### 2.5.1 fNIRS data processing

The fNIRS data were processed using Homer2 in MATLAB R2013b. Raw light intensity signals were visually inspected for saturation, flat signals, discontinuities, and persistent poor optical quality. All channels met the predefined quality criteria. The remaining signals were converted into optical density data. Motion artifacts were identified using a 0.5-s moving window, with changes exceeding 10 standard deviations defined as artifacts, and corrected using spline interpolation with the Homer2 function hmrMotionCorrectSpline (*p* = 0.99). No channels or complete task blocks were excluded from any group. The corrected signals were band-pass filtered at 0.01–0.1 Hz and converted to hemoglobin concentration changes using the modified Beer–Lambert law (29). For each block, the mean HbO value during the final 5 s of the immediately preceding resting period was used as the baseline. Task-related HbO responses were calculated relative to this baseline and averaged separately for the high-sweetness food, low-sweetness food, and non-food control conditions. For each participant, time point, and condition, valid channels within each ROI were averaged to obtain the mean ROI-level HbO response.

#### 2.5.2 Statistical analysis

All statistical analyses were performed using IBM SPSS 26.0. Continuous variables were expressed as mean ± standard deviation. Normality and homogeneity of variance were assessed using the Shapiro–Wilk test and Levene’s test, respectively. A 3 × 2 mixed-design ANOVA was used to examine the effects of group, time, and their interaction on VAS scores and HbO values in PFC ROIs under the three VFCP conditions. Bonferroni correction was applied for *post hoc* comparisons of behavioral outcomes. For HbO analyses, false discovery rate correction was applied across 24 ROI-condition combinations, corresponding to 8 PFC ROIs under 3 VFCP conditions.

Changes in VAS scores and HbO values were calculated as post-intervention minus pre-intervention values. Correlation analysis was performed to examine associations between changes in sweetness-related food preference and changes in PFC HbO. Mediation analysis was then conducted to explore whether changes in PFC HbO mediated the effect of acute aerobic exercise on sweetness-related food preference. Indirect effects were tested using 5,000 bootstrap samples, and mediation was considered significant when the 95% confidence interval did not include zero. The significance level was set at *p* < 0.05.

## 3 Results

### 3.1 Sweetness-related food preference scores

**Figure 5:**
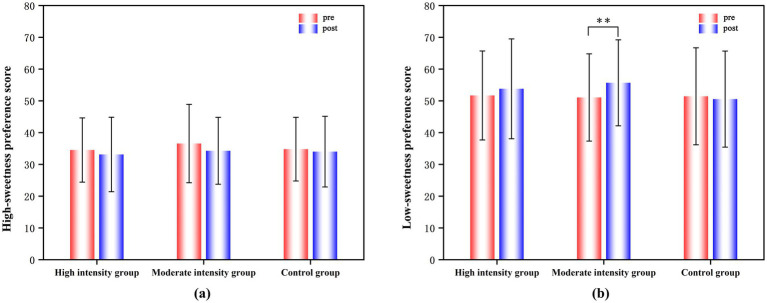
Preference scores of participants in the three groups at pre- and post-intervention. **(a)** Preference scores for low-sweetness foods; **(b)** preference scores for high-sweetness foods. **p < 0.01. Pre denotes pre-intervention test; post denotes post-intervention test.

For low-sweetness food preference, a significant group × time interaction [*F*_(2, 57)_ = 5.345, *p* = 0.008, *ηp^2^* = 0.158] and a significant main effect of time [*F*_(1, 57)_ = 7.662, *p* = 0.008, *ηp^2^* = 0.118] were observed, with the overall mean score increasing from pre- to post-intervention (51.40 vs. 53.68). The interaction was characterized by a greater increase in the moderate-intensity exercise group (51.06 ± 13.73 vs. 56.69 ± 13.53), a smaller increase in the high-intensity exercise group (51.70 ± 14.01 vs. 53.80 ± 15.72), and a slight decrease in the rest control group (51.43 ± 15.26 vs. 50.55 ± 15.12). Further simple-effects analysis showed that the pre–post increase was significant only in the moderate-intensity exercise group (*p* < 0.001) (Tables 4, 5, Figure 5).

**Table 4:** Descriptive statistics for low- and high-sweetness food preference scores.

Group	Low-sweetness		High-sweetness	
	Pre-test	Post-test	Pre-test	Post-test
High-intensity	51.70 ± 14.01	53.80 ± 15.72	35.20 ± 15.31	30.10 ± 12.44
Moderate-intensity	51.06 ± 13.73	56.69 ± 13.53	46.57 ± 16.53	43.42 ± 20.58
Control	51.43 ± 15.26	50.55 ± 15.12	34.81 ± 19.04	34.03 ± 19.33

**Table 5:** Mixed-design ANOVA results for low- and high-sweetness food preference scores.

Effect	Low-sweetness				High-sweetness			
	*F*	*p*	ηp^2^	[95% CI]	*F*	*p*	ηp^2^	[95% CI]
Time effect	7.662	0.008	0.118	[0.009, 0.286]	5.321	0.025	0.085	[0.000, 0.245]
Group effect	0.142	0.868	0.005	[0.000, 0.059]	2.767	0.072	0.088	[0.000, 0.236]
Interaction effect	5.345	0.008	0.158	[0.014, 0.321]	0.956	0.391	0.032	[0.000, 0.145]

### 3.2 Activation of the PFC

A 3 × 2 mixed-design ANOVA was conducted to examine HbO responses in the PFC ROIs. Under the low-sweetness food condition, significant main effects of time were observed in the R-VLPFC [*F*_(1, 57)_ = 4.657, *p* = 0.035, *ηp^2^* = 0.076], L-FP [*F*_(1, 57)_ = 9.388, *p* = 0.003, *ηp^2^* = 0.141], R-FP [*F*_(1, 57)_ = 8.944, *p* = 0.004, *ηp^2^* = 0.136], L-OFC [*F*_(1, 57)_ = 8.115, *p* = 0.006, *ηp^2^* = 0.125], and R-OFC [*F*_(1, 57)_ = 8.134, *p* = 0.006, *ηp^2^* = 0.125]. Across groups, HbO responses decreased from pre- to post-intervention in the R-VLPFC (1.09 vs. −0.63), L-FP (0.76 vs. −0.78), R-FP (0.45 vs. −0.92), and L-OFC (1.01 vs. −1.16), whereas the R-OFC response increased (−1.17 vs. 0.89).

**Figure 6:**
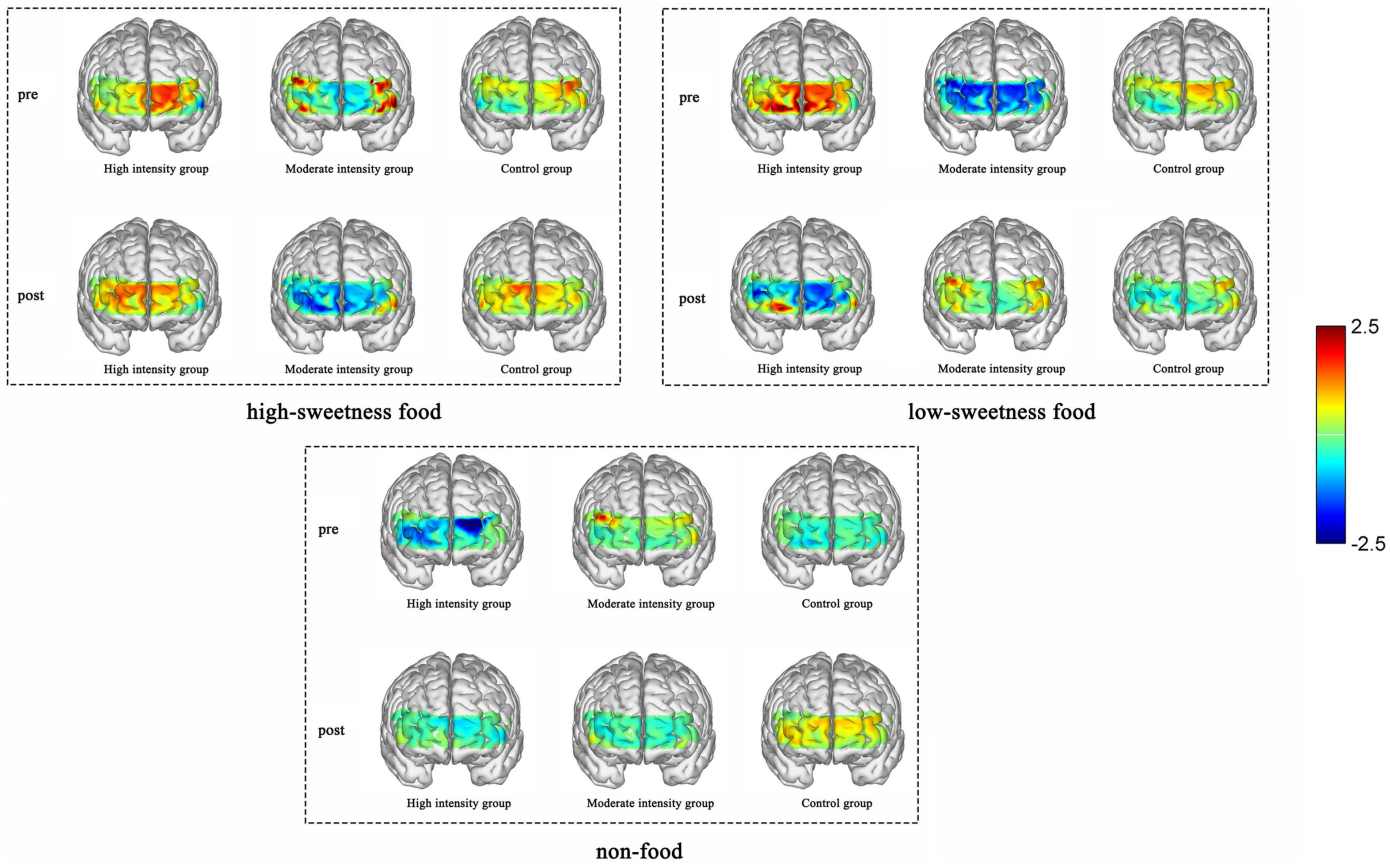
Mean PFC HbO concentrations at pre- and post-intervention among the three participant groups under different stimuli (×10^−7^ mmol).

**Figure 7:**
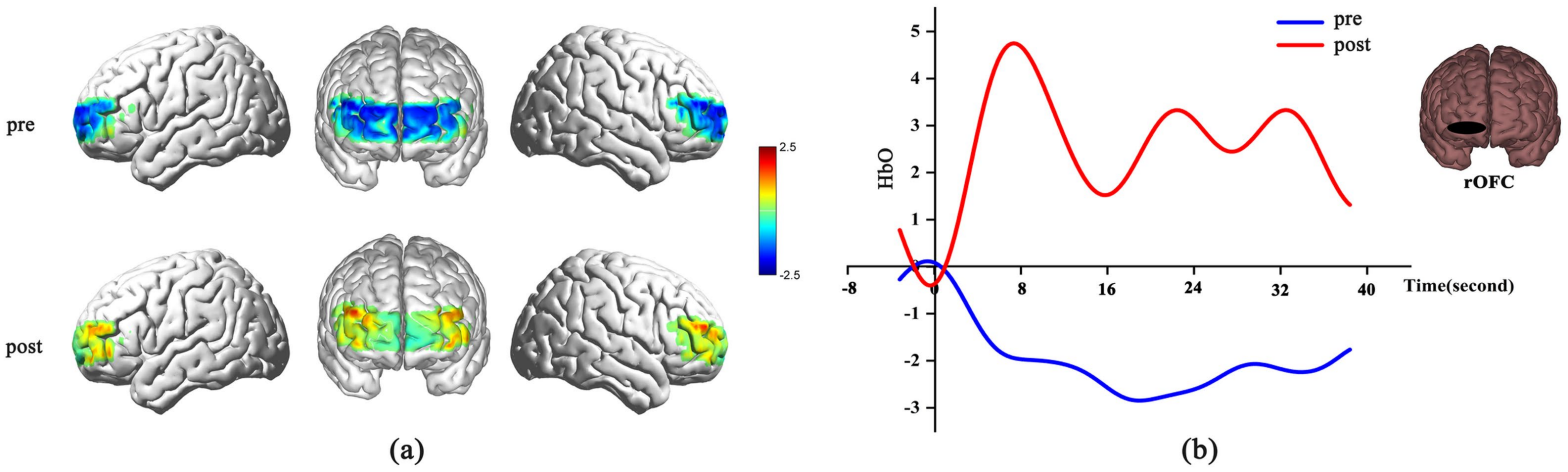
Changes in HbO concentration in response to low-sweetness food stimulation at pre- and post-intervention in the moderate-intensity exercise group (×10^−7^ mmol). **(a)** Mean HbO responses in the PFC; **(b)** time course of R-OFC HbO responses during the low-sweetness food cue task. Pre denotes pre-intervention test; post denotes post-intervention test.

Only the R-OFC showed a significant group × time interaction [*F*_(2, 57)_ = 4.220, *p* = 0.020, *ηp^2^* = 0.129]. The interaction was characterized by a marked increase in the moderate-intensity exercise group (−2.07 ± 2.52 vs. 2.68 ± 6.99), a smaller increase in the high-intensity exercise group (−1.49 ± 3.30 vs. 0.34 ± 3.05), and a slight decrease in the rest control group (0.06 ± 3.67 vs. −0.35 ± 2.96). Further simple-effects analysis showed that the pre–post increase was significant only in the moderate-intensity exercise group (*p* < 0.001). No significant group × time interactions were observed in the other PFC ROIs or under the high-sweetness food and non-food conditions (Tables 6–11; Figures 6, 7).

**Table 6:** Descriptive statistics for PFC HbO responses under the low-sweetness food condition.

ROI	High-intensity		Moderate-intensity		Control	
	Pre-test	Post-test	Pre-test	Post-test	Pre-test	Post-test
L-DLPFC	0.62 ± 5.78	0.25 ± 4.88	1.60 ± 6.31	−1.94 ± 4.09	0.73 ± 3.87	1.31 ± 4.94
R-DLPFC	0.17 ± 6.22	1.85 ± 8.59	1.70 ± 5.54	−0.19 ± 9.41	0.93 ± 11.49	2.30 ± 7.28
L-VLPFC	−0.04 ± 4.72	1.17 ± 5.10	1.06 ± 3.22	−0.29 ± 3.69	0.38 ± 6.54	1.01 ± 3.67
R-VLPFC	1.92 ± 5.55	0.09 ± 4.35	0.61 ± 3.82	−1.29 ± 2.14	0.74 ± 3.84	−0.68 ± 5.13
L-FP	1.06 ± 3.70	−1.03 ± 2.34	0.30 ± 3.20	−1.55 ± 2.09	0.92 ± 3.03	0.23 ± 2.99
R-FP	0.64 ± 3.19	−0.96 ± 2.67	0.32 ± 2.83	−1.64 ± 1.97	0.38 ± 2.24	−0.16 ± 2.98
L-OFC	2.36 ± 7.11	−1.56 ± 2.75	0.51 ± 4.20	−1.69 ± 3.27	0.16 ± 4.10	−0.24 ± 3.03
R-OFC	−1.49 ± 3.30	0.34 ± 3.05	−2.07 ± 2.52	2.68 ± 6.99	0.06 ± 3.67	−0.35 ± 2.96

OFC denotes the orbitofrontal cortex; DLPFC denotes the dorsolateral prefrontal cortex; VLPFC denotes the ventrolateral prefrontal cortex; FP denotes the frontal pole; L indicates the left hemisphere; R indicates the right hemisphere; pre denotes pre-intervention test; post denotes post-intervention test.

**Table 7:** Mixed-design ANOVA results and effect sizes for PFC HbO responses under the low-sweetness food condition.

ROI	Effect	*F*	*p*	ηp^2^	95% CI
L-DLPFC	Time effect	1.593	0.212	0.027	[0.000, 0.155]
	Group effect	0.480	0.621	0.017	[0.000, 0.107]
	Interaction effect	2.032	0.141	0.067	[0.000, 0.204]
R-DLPFC	Time effect	0.077	0.782	0.001	[0.000, 0.072]
	Group effect	0.092	0.912	0.003	[0.000, 0.044]
	Interaction effect	0.683	0.509	0.023	[0.000, 0.125]
L-VLPFC	Time effect	0.042	0.838	0.001	[0.000, 0.062]
	Group effect	0.038	0.963	0.001	[0.000, 0.014]
	Interaction effect	0.998	0.375	0.034	[0.000, 0.148]
R-VLPFC	Time effect	4.657	0.035	0.076	[0.000, 0.232]
	Group effect	1.040	0.360	0.035	[0.000, 0.150]
	Interaction effect	0.035	0.965	0.001	[0.000, 0.011]
L-FP	Time effect	9.388	0.003	0.141	[0.018, 0.313]
	Group effect	1.409	0.253	0.047	[0.000, 0.172]
	Interaction effect	0.727	0.488	0.025	[0.000, 0.128]
R-FP	Time effect	8.944	0.004	0.136	[0.015, 0.306]
	Group effect	0.716	0.493	0.025	[0.000, 0.128]
	Interaction effect	0.880	0.421	0.030	[0.000, 0.140]
L-OFC	Time effect	8.115	0.006	0.125	[0.011, 0.293]
	Group effect	0.471	0.627	0.016	[0.000, 0.106]
	Interaction effect	1.755	0.182	0.058	[0.000, 0.191]
R-OFC	Time effect	8.134	0.006	0.125	[0.011, 0.294]
	Group effect	0.438	0.647	0.015	[0.000, 0.103]
	Interaction effect	4.220	0.020	0.129	[0.003, 0.287]

OFC denotes the orbitofrontal cortex; DLPFC denotes the dorsolateral prefrontal cortex; VLPFC denotes the ventrolateral prefrontal cortex; FP denotes the frontal pole; L indicates the left hemisphere; R indicates the right hemisphere; pre denotes pre-intervention test; post denotes post-intervention test.

**Table 8:** Descriptive statistics for PFC HbO responses under the high-sweetness food condition.

ROI	High-intensity		Moderate-intensity		Control	
	Pre-test	Post-test	Pre-test	Post-test	Pre-test	Post-test
L-DLPFC	1.48 ± 4.82	1.10 ± 7.38	1.72 ± 6.77	1.13 ± 10.72	1.17 ± 4.06	0.42 ± 4.11
R-DLPFC	−1.27 ± 8.88	−0.59 ± 6.47	5.14 ± 12.80	−1.80 ± 13.80	1.54 ± 9.54	0.10 ± 8.86
L-VLPFC	−1.12 ± 4.01	−0.27 ± 6.51	3.08 ± 4.69	1.02 ± 8.77	1.12 ± 6.82	0.36 ± 4.49
R-VLPFC	1.33 ± 5.68	0.97 ± 5.49	0.85 ± 3.93	−0.67 ± 5.00	−0.61 ± 4.70	1.49 ± 3.01
L-FP	1.18 ± 3.46	0.57 ± 3.48	0.42 ± 3.52	−1.03 ± 3.32	0.75 ± 2.33	0.82 ± 2.17
R-FP	0.43 ± 3.01	1.32 ± 4.39	−0.27 ± 2.73	−0.97 ± 3.04	0.34 ± 2.95	0.84 ± 1.93
L-OFC	0.91 ± 7.18	0.66 ± 5.31	0.86 ± 3.90	−0.42 ± 5.48	0.27 ± 4.38	0.47 ± 2.36
R-OFC	0.91 ± 6.37	0.89 ± 5.06	1.17 ± 4.66	−1.61 ± 4.95	−0.03 ± 3.38	0.51 ± 2.38

OFC denotes the orbitofrontal cortex; DLPFC denotes the dorsolateral prefrontal cortex; VLPFC denotes the ventrolateral prefrontal cortex; FP denotes the frontal pole; L indicates the left hemisphere; R indicates the right hemisphere; pre denotes pre-intervention test; post denotes post-intervention test.

**Table 9:** Mixed-design ANOVA results and effect sizes for PFC HbO responses under the high-sweetness food condition.

ROI	Effect	*F*	*p*	ηp^2^	95% CI
L-DLPFC	Time effect	0.259	0.613	0.005	[0.000, 0.095]
	Group effect	0.077	0.926	0.003	[0.000, 0.038]
	Interaction effect	0.009	0.991	0.000	[0.000, 0.000]
R-DLPFC	Time effect	2.019	0.161	0.034	[0.000, 0.168]
	Group effect	0.555	0.577	0.019	[0.000, 0.114]
	Interaction effect	1.600	0.211	0.053	[0.000, 0.183]
L-VLPFC	Time effect	0.379	0.541	0.007	[0.000, 0.103]
	Group effect	1.759	0.182	0.058	[0.000, 0.191]
	Interaction effect	0.619	0.542	0.021	[0.000, 0.120]
R-VLPFC	Time effect	0.008	0.930	0.000	[0.000, 0.035]
	Group effect	0.483	0.619	0.017	[0.000, 0.107]
	Interaction effect	1.539	0.224	0.051	[0.000, 0.180]
L-FP	Time effect	1.332	0.253	0.023	[0.000, 0.146]
	Group effect	1.764	0.181	0.058	[0.000, 0.191]
	Interaction effect	0.594	0.556	0.020	[0.000, 0.118]
R-FP	Time effect	0.159	0.692	0.003	[0.000, 0.085]
	Group effect	2.663	0.079	0.085	[0.000, 0.231]
	Interaction effect	0.688	0.507	0.024	[0.000, 0.125]
L-OFC	Time effect	0.257	0.614	0.004	[0.000, 0.095]
	Group effect	0.118	0.889	0.004	[0.000, 0.053]
	Interaction effect	0.252	0.778	0.009	[0.000, 0.080]
R-OFC	Time effect	0.724	0.399	0.013	[0.000, 0.122]
	Group effect	0.604	0.550	0.021	[0.000, 0.118]
	Interaction effect	1.348	0.268	0.045	[0.000, 0.169]

OFC denotes the orbitofrontal cortex; DLPFC denotes the dorsolateral prefrontal cortex; VLPFC denotes the ventrolateral prefrontal cortex; FP denotes the frontal pole; L indicates the left hemisphere; R indicates the right hemisphere; pre denotes pre-intervention test; post denotes post-intervention test.

**Table 10:** Descriptive statistics for PFC HbO responses under the non-food condition.

ROI	High-intensity		Moderate-intensity		Control	
	Pre-test	Post-test	Pre-test	Post-test	Pre-test	Post-test
L-DLPFC	0.09 ± 4.65	0.16 ± 4.17	1.50 ± 4.71	0.05 ± 5.96	−0.13 ± 4.47	0.71 ± 2.59
R-DLPFC	1.05 ± 7.77	−1.04 ± 4.30	0.73 ± 4.89	0.36 ± 6.00	−0.19 ± 8.62	−0.51 ± 5.24
L-VLPFC	−0.16 ± 3.59	−1.47 ± 2.42	0.90 ± 4.03	−0.14 ± 2.74	−0.43 ± 4.51	0.74 ± 3.24
R-VLPFC	−1.02 ± 4.39	0.31 ± 4.18	0.10 ± 3.55	0.29 ± 2.77	0.20 ± 3.92	0.77 ± 3.25
L-FP	−0.22 ± 3.52	−0.63 ± 2.22	0.35 ± 2.58	−0.23 ± 2.28	−0.22 ± 2.61	1.00 ± 2.57
R-FP	−1.11 ± 3.87	−0.41 ± 3.13	0.25 ± 1.96	−0.50 ± 1.54	−0.06 ± 2.34	0.43 ± 2.58
L-OFC	−0.18 ± 5.05	0.03 ± 3.44	−0.36 ± 2.97	0.08 ± 2.39	−0.81 ± 4.41	0.39 ± 2.89
R-OFC	−0.49 ± 5.14	0.14 ± 3.35	−0.16 ± 2.43	−0.12 ± 3.19	−1.04 ± 3.18	0.74 ± 3.11

OFC denotes the orbitofrontal cortex; DLPFC denotes the dorsolateral prefrontal cortex; VLPFC denotes the ventrolateral prefrontal cortex; FP denotes the frontal pole; L indicates the left hemisphere; R indicates the right hemisphere; pre denotes pre-intervention test; post denotes post-intervention test.

**Table 11:** Mixed-design ANOVA results and effect sizes for PFC HbO responses under the non-food condition.

ROI	Effect	*F*	*p*	ηp^2^	95% CI
L-DLPFC	Time effect	0.043	0.836	0.001	[0.000, 0.063]
	Group effect	0.222	0.801	0.008	[0.000, 0.075]
	Interaction effect	0.627	0.538	0.022	[0.000, 0.120]
R-DLPFC	Time effect	0.707	0.404	0.012	[0.000, 0.121]
	Group effect	0.178	0.837	0.006	[0.000, 0.067]
	Interaction effect	0.279	0.758	0.010	[0.000, 0.084]
L-VLPFC	Time effect	0.396	0.532	0.007	[0.000, 0.104]
	Group effect	1.165	0.320	0.039	[0.000, 0.158]
	Interaction effect	1.588	0.213	0.053	[0.000, 0.182]
R-VLPFC	Time effect	1.169	0.284	0.020	[0.000, 0.140]
	Group effect	0.451	0.639	0.016	[0.000, 0.104]
	Interaction effect	0.269	0.765	0.009	[0.000, 0.083]
L-FP	Time effect	0.026	0.872	0.000	[0.000, 0.055]
	Group effect	0.860	0.429	0.029	[0.000, 0.138]
	Interaction effect	1.400	0.255	0.047	[0.000, 0.172]
R-FP	Time effect	0.089	0.767	0.002	[0.000, 0.075]
	Group effect	1.222	0.303	0.041	[0.000, 0.162]
	Interaction effect	0.860	0.429	0.029	[0.000, 0.138]
L-OFC	Time effect	0.877	0.353	0.015	[0.000, 0.128]
	Group effect	0.013	0.988	0.000	[0.000, 0.000]
	Interaction effect	0.202	0.817	0.007	[0.000, 0.072]
R-OFC	Time effect	1.986	0.164	0.034	[0.000, 0.167]
	Group effect	0.001	0.999	0.000	[0.000, 0.000]
	Interaction effect	0.775	0.466	0.026	[0.000, 0.132]

OFC denotes the orbitofrontal cortex; DLPFC denotes the dorsolateral prefrontal cortex; VLPFC denotes the ventrolateral prefrontal cortex; FP denotes the frontal pole; L indicates the left hemisphere; R indicates the right hemisphere; pre denotes pre-intervention test; post denotes post-intervention test.

### 3.3 Correlation analysis

Pearson correlation analysis was performed to examine the association between changes in VAS scores and changes in R-OFC HbO responses. The results showed that, in the moderate-intensity exercise group, changes in VAS scores for low-sweetness food images were significantly positively correlated with changes in R-OFC HbO under the low-sweetness food condition (*r* = 0.835, *p* < 0.001). No significant correlations were observed under the high-sweetness food condition or in the rest control group (Table 12).

**Table 12:** Correlations between changes in sweetness-related food preference scores and R-OFC HbO responses.

Group	Low-sweetness			High-sweetness		
	*r*	*p*	95% CI	*r*	*p*	95% CI
Moderate-intensity	0.835	<0.001	[0.622, 0.933]	0.175	0.502	[−0.290, 0.573]
Resting control	0.320	0.181	[−0.143, 0.668]	0.155	0.526	[−0.309, 0.559]

Changes were calculated as post-intervention minus pre-intervention values.

### 3.4 Exploratory mediation analysis

Regression analyses showed that moderate-intensity exercise significantly predicted changes in low-sweetness food preference scores (*t* = 8.814, *p* < 0.001). After changes in R-OFC HbO were introduced as a mediator, moderate-intensity exercise significantly predicted changes in R-OFC HbO under the low-sweetness food condition (*t* = 8.174, *p* < 0.001). In turn, changes in R-OFC HbO significantly predicted changes in low-sweetness food preference scores (*t* = 7.387, *p* < 0.001). After the mediator was included in the model, the direct effect of moderate-intensity exercise on low-sweetness food preference remained significant (*t* = 2.202, *p* = 0.035).

**Figure 8:**
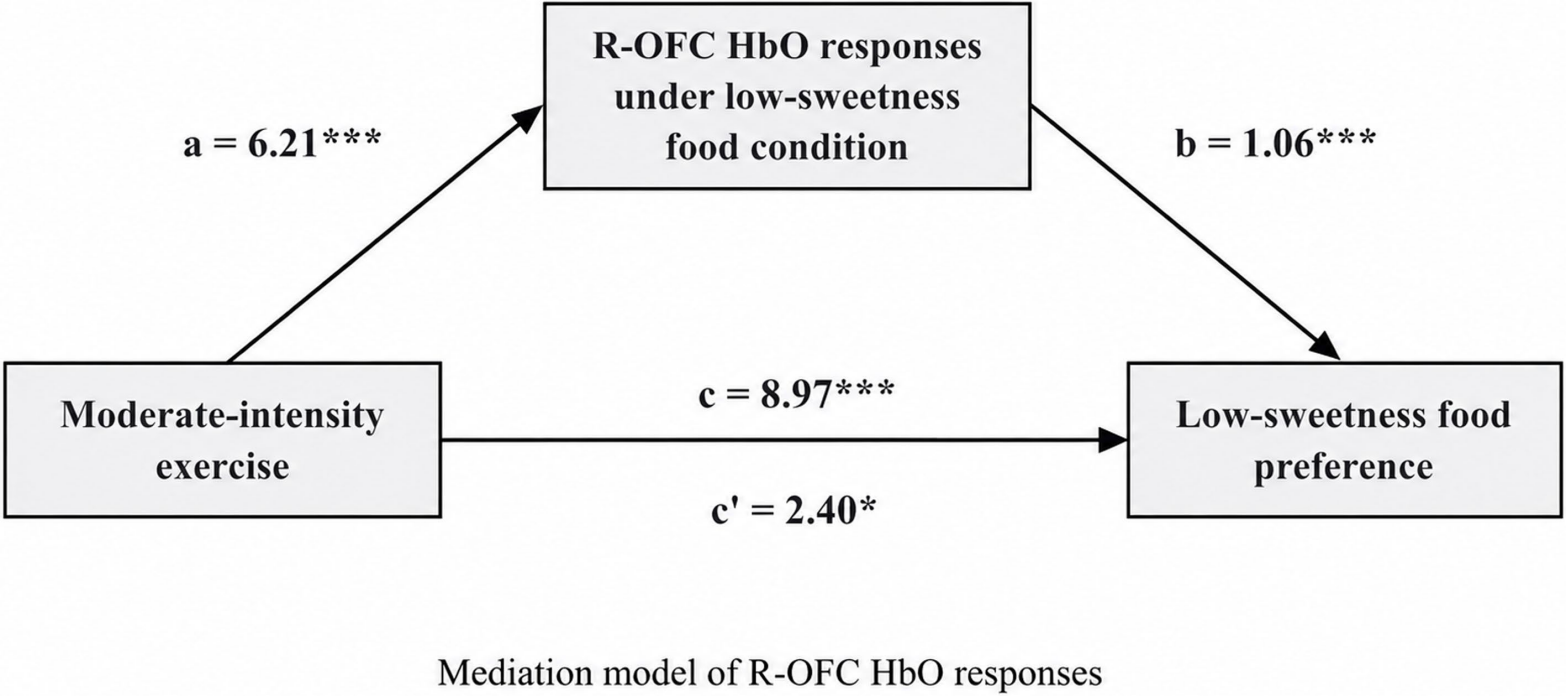
Mediation model of R-OFC HbO responses in the effect of moderate-intensity exercise on low-sweetness food preference. *p < 0.05, **p < 0.001.

Bootstrap analysis further showed that the 95% confidence interval for the indirect effect did not include zero, indicating a significant indirect effect (Figure 8). These results indicate an exploratory statistical mediation pattern involving changes in R-OFC HbO responses in the association between moderate-intensity exercise and increased low-sweetness food preference (Table 13).

**Table 13:** Mediation analysis of R-OFC HbO responses in the relationship between moderate-intensity exercise and low-sweetness food preference.

Effect pathway	B	SE	*p*	95% CI
a: X → M	6.21	0.76	<0.001	[4.66, 7.75]
b: M → Y	1.06	0.14	<0.001	[0.77, 1.35]
c: X → Y, total effect	8.97	1.02	<0.001	[6.90, 11.04]
c’: X → Y, direct effect	2.40	1.09	0.035	[0.18, 4.62]
a × b: indirect effect	6.56	1.23	-	[4.29, 9.13]

X was coded as 1 for the moderate-intensity exercise group and 0 for the rest control group. M represents changes in R-OFC HbO responses under the low-sweetness food condition, and Y represents changes in VAS preference scores for low-sweetness food cues. Changes were calculated as post-intervention minus pre-intervention values.

## 4 Discussion

In the present study, sweetness-related food preference refers to the immediate self-reported tendency to choose visually presented foods differing in perceived sweetness and may reflect subjective responsiveness to food-related natural reward cues (30). Because participants did not actually taste or consume the foods, this measure should be interpreted as image-based food preference rather than gustatory sweetness-related food preference. HbO responses reflect changes in prefrontal cortical hemodynamic activity elicited by visual food cues (31). Such pre-consumption cue evaluation may capture an early stage before food selection, as visual cues can shape food-related decisions before eating, although it cannot be equated with actual intake. Given that individuals with nicotine dependence (ND) may exhibit abnormalities in reward processing (31, 32), we further examined whether acute aerobic exercise modulates sweetness-related food preference and whether these behavioral changes are associated with prefrontal cortical activity. The results showed that moderate-intensity exercise had more pronounced effects on low-sweetness food-cue preference and R-OFC HbO responses.

Notably, changes in sweetness-related food preference were primarily observed for low-sweetness rather than high-sweetness food cues. This category-specific pattern may reflect differences in reward salience. In individuals with ND, chronic nicotine exposure may increase sensitivity to highly salient and immediately reinforcing stimuli, potentially maintaining relatively strong responses to high-sweetness food cues (7, 10, 11). In contrast, low-sweetness food cues may have relatively lower reward salience and may therefore be more sensitive to transient exercise-related changes in food-cue evaluation (33). The increased preference for low-sweetness food cues following moderate-intensity exercise may therefore be consistent with a short-term increase in responsiveness to food cues with relatively lower reward salience, rather than a general increase in appetite or sweetness preference. This cue-level shift may represent an early change in food-choice evaluation, although its translation into actual food selection or consumption in naturalistic settings requires further investigation.

From a neurobiological perspective, the R-OFC may be involved in how moderate-intensity exercise modulates preference for low-sweetness food cues. As a central component of the prefrontal reward network, the OFC integrates multimodal sensory information from gustatory, olfactory, and visual food cues and dynamically evaluates the hedonic value, reward significance, and subjective attractiveness of food stimuli (34, 35). In individuals with ND, chronic nicotine exposure may increase sensitivity to highly salient and immediate rewards while reducing responsiveness to relatively mild natural rewards (36, 37). In the present study, moderate-intensity exercise enhanced R-OFC HbO responses elicited by low-sweetness food cues, and this enhancement was significantly associated with increased preference for low-sweetness food cues. These findings suggest that exercise may strengthen R-OFC involvement in the valuation of low-sweetness food cues. In other words, moderate-intensity exercise may not simply amplify responsiveness to sweet food cues, but rather may modulate OFC-mediated valuation of food cues, thereby increasing the subjective value of low-sweetness food cues during behavioral evaluation (38, 39). The exploratory mediation analysis further showed a significant indirect effect, indicating a statistical mediation pattern involving changes in R-OFC HbO responses in the association between moderate-intensity exercise and increased preference for low-sweetness food cues. Taken together, these findings support the R-OFC as a potential neural correlate of the association between acute exercise and changes in the evaluation of low-sweetness food cues in individuals with ND.

Furthermore, the present study found that high-intensity exercise did not produce modulatory effects comparable to those observed after moderate-intensity exercise, suggesting that exercise intensity may be an important factor influencing sweetness-related food-cue preference. Moderate-intensity exercise may enhance physiological arousal and prefrontal regulatory function while avoiding excessive fatigue or stress responses, thereby facilitating the evaluation of food cues (40, 41). In contrast, high-intensity exercise may transiently increase physiological load and alter attentional resources, affective state, and homeostatic regulatory demands, which could attenuate the effects observed for low-sweetness food-cue preference (42–44). These findings suggest that the effect of acute aerobic exercise on sweetness-related food preference in individuals with ND may not follow a simple “higher intensity, greater effect” pattern, but instead may depend on an optimal intensity range (45–47). From a behavioral-intervention perspective, modulating food-cue evaluation before consumption may help influence the early decision stage preceding food choice. Thus, appropriately prescribed acute exercise may be considered as a potential timing-specific strategy before food-cue exposure or dietary decision-making, although its effects on actual food choice and intake require further investigation.

Although the present study provides preliminary behavioral and neurophysiological evidence regarding the effects of moderate-intensity acute aerobic exercise on low-sweetness food preference in individuals with ND, several limitations should be acknowledged. First, the sample included only male university students aged 18–25 years, limiting the generalizability of the findings to females and other age groups. Second, subjective hunger, satiety, cigarette craving, withdrawal symptoms, and mood were not assessed; therefore, individual differences in these states may have influenced food-cue preference and prefrontal hemodynamic responses despite standardized fasting and smoking-abstinence requirements. Third, the study examined only the immediate effects of a single exercise session, and sweetness-related food preference was assessed using self-reported ratings of visual food cues rather than actual food selection or consumption. In addition, because stimuli were classified by perceived sweetness without independently manipulating or matching caloric density, the observed effects cannot be attributed exclusively to sweetness. The exploratory mediation analysis should also be confirmed in larger studies specifically powered to evaluate this pathway. Finally, objective attention monitoring, concurrent physiological measurements, and short-separation fNIRS channels were not included; thus, contributions from general arousal, visual attention, and residual superficial or systemic hemodynamic changes to R-OFC HbO responses cannot be fully excluded. Future studies with more diverse samples, objective food-choice measures, and improved physiological and fNIRS controls are needed to confirm and extend these findings.

Taken together, the present findings suggest that acute aerobic exercise may influence sweetness-related food-cue evaluation in individuals with ND. Moderate-intensity exercise was associated with increased preference for low-sweetness food cues and enhanced R-OFC hemodynamic responses, indicating a potential link between exercise-induced changes in the evaluation of low-sweetness food cues and prefrontal function. These findings provide preliminary evidence that acute exercise may affect responses to non-drug food cues in ND and provide a basis for future studies examining whether such short-term neurobehavioral changes translate into meaningful dietary or smoking-related outcomes.

## 5 Conclusion

Acute aerobic exercise exerted intensity-dependent effects on sweetness-related food preference in individuals with ND. Specifically, moderate-intensity exercise significantly increased preference for low-sweetness food cues, whereas high-intensity exercise showed no significant effect. In addition, exploratory mediation analysis identified a significant indirect effect through changes in R-OFC HbO responses under the low-sweetness condition in the association between moderate-intensity exercise and changes in preference for low-sweetness food cues. These findings suggest that moderate-intensity acute aerobic exercise may represent a promising approach for modulating the evaluation of low-sweetness food cues in individuals with ND and provide preliminary evidence for the potential role of exercise in influencing sweetness-related food-cue evaluation.

## Data Availability

The original contributions presented in the study are included in the article/supplementary material, further inquiries can be directed to the corresponding author.

## References

[1] Colyer-Patel K Kuhns L Weidema A Lesscher H Cousijn J. Age-dependent effects of tobacco smoke and nicotine on cognition and the brain: a systematic review of the human and animal literature comparing adolescents and adults. Neurosci Biobehav Rev. (2023) 146:105038. doi: 10.1016/j.neubiorev.2023.105038, 36627063

[2] Wills L Ables JL Braunscheidel KM Caligiuri SPB Elayouby KS Fillinger C . Neurobiological mechanisms of nicotine reward and aversion. Pharmacol Rev. (2022) 74:271–310. doi: 10.1124/pharmrev.121.000299, 35017179 PMC11060337

[3] Hahad O Daiber A Michal M Kuntic M Lieb K Beutel M . Smoking and neuropsychiatric disease-associations and underlying mechanisms. Int J Mol Sci. (2021) 22:7272. doi: 10.3390/ijms22147272, 34298890 PMC8304236

[4] Small DM Veldhuizen MG Felsted J Mak YE McGlone F. Separable substrates for anticipatory and consummatory food chemosensation. Neuron. (2008) 57:786–97. doi: 10.1016/j.neuron.2008.01.021, 18341997 PMC2669434

[5] Veldhuizen MG Babbs RK Patel B Fobbs W Kroemer NB Garcia E . Integration of sweet taste and metabolism determines carbohydrate reward. Curr Biol. (2017) 27:2476–2485.e6. doi: 10.1016/j.cub.2017.07.018, 28803868 PMC5745144

[6] Liu H Zhang Y Zhang S Xu Z. Effects of acute aerobic exercise on food-reward mechanisms in smoking-addicted individuals: an fNIRS study. Physiol Behav. (2022) 254:113889. doi: 10.1016/j.physbeh.2022.113889, 35738424

[7] Anker JJ Nakajima M Raatz S Allen S al’Absi M. Tobacco withdrawal increases junk food intake: the role of the endogenous opioid system. Drug Alcohol Depend. (2021) 225:108819. doi: 10.1016/j.drugalcdep.2021.108819, 34182373 PMC8297656

[8] Alruwaili A King JA Deighton K Kelly BM Liao Z Innes A . The association of smoking with different eating and dietary behaviours: a cross-sectional analysis of 80 296 United Kingdom adults. Addiction. (2024) 119:1737–50. doi: 10.1111/add.16584, 38884138

[9] Anker JJ al'Absi M. Effects of opioid blockade on taste perception across smoking status: an analysis of detection thresholds, intensity, and pleasantness. J Neural Transm. (2025) 132:1411–6. doi: 10.1007/s00702-025-02937-9, 40944723 PMC12535516

[10] Alruwaili A Nayeemullah R Engin B Malaikah S James L Sanders JP . The association of cigarette smoking with appetite, appetite-related hormones and food reward: a matched-pair cohort study. Appetite. (2025) 214:108194. doi: 10.1016/j.appet.2025.108194, 40513832

[11] van Amsterdam J van den Brink W. Sweet-liking and sugar supplementation as innovative components in substance use disorder treatment: a systematic review. J Psychopharmacol. (2025) 39:328–38. doi: 10.1177/02698811251319454, 39945416 PMC11963440

[12] Cheung YT Lam TH Chan CHH Ho KS Fok WYP Wang MP . Brief handgrip and isometric exercise intervention for smoking cessation: a pilot randomized trial. Addict Behav. (2020) 100:106119. doi: 10.1016/j.addbeh.2019.106119, 31522134

[13] Sanchez V Bakhti-Suroosh A Chen A Brunzell DH Erisir A Lynch WJ. Exercise during abstinence normalizes ultrastructural synaptic plasticity associated with nicotine-seeking following extended access self-administration. Eur J Neurosci. (2019) 50:2707–21. doi: 10.1111/ejn.14408, 30888721 PMC6742551

[14] Taylor AH Ussher MH Faulkner G. The acute effects of exercise on cigarette cravings, withdrawal symptoms, affect and smoking behaviour: a systematic review. Addiction. (2007) 102:534–43. doi: 10.1111/j.1360-0443.2006.01739.x, 17286639

[15] Gorrell S Shott ME Frank GKW. Associations between aerobic exercise and dopamine-related reward-processing: informing a model of human exercise engagement. Biol Psychol. (2022) 171:108350. doi: 10.1016/j.biopsycho.2022.108350, 35561818 PMC9869713

[16] Zhou YU Finlayson G Liu X Zhou Q Liu T Zhou C. Effects of acute dance and aerobic exercise on drug craving and food reward in women with methamphetamine dependence. Med Sci Sports Exerc. (2021) 53:2245–53. doi: 10.1249/MSS.0000000000002723, 34115731

[17] Mekari S Fraser S Bosquet L Bonnéry C Labelle V Pouliot P . The relationship between exercise intensity, cerebral oxygenation and cognitive performance in young adults. Eur J Appl Physiol. (2015) 115:2189–97. doi: 10.1007/s00421-015-3199-4, 26063061

[18] Moriarty T Bourbeau K Bellovary B Zuhl MN. Exercise intensity influences prefrontal cortex oxygenation during cognitive testing. Behav Sci. (2019) 9:83. doi: 10.3390/bs9080083, 31357450 PMC6721405

[19] Kunicki ZJ Hallgren M Uebelacker LA Brown RA Price LH Abrantes AM. Examining the effect of exercise on the relationship between affect and cravings among smokers engaged in cessation treatment. Addict Behav. (2022) 125:107156. doi: 10.1016/j.addbeh.2021.107156, 34710842 PMC8629942

[20] Zhou Y Feng W Guo Y Wu J. Effect of exercise intervention on smoking cessation: a meta-analysis. Front Physiol. (2023) 14:1221898. doi: 10.3389/fphys.2023.1221898, 37614760 PMC10442508

[21] Ceceli AO Bradberry CW Goldstein RZ. The neurobiology of drug addiction: cross-species insights into the dysfunction and recovery of the prefrontal cortex. Neuropsychopharmacology. (2022) 47:276–91. doi: 10.1038/s41386-021-01153-9, 34408275 PMC8617203

[22] Murray L Scavnicky MK Korponay C Lukas SE Frederick BB Janes AC. Brain reactivity to nicotine cues mediates the link between resting-state connectivity and cue-induced craving in individuals who smoke or vape nicotine. Neuropsychopharmacology. (2025) 50:983–90. doi: 10.1038/s41386-025-02083-6, 40082646 PMC12032118

[23] Mason TB Dolgon-Krutolow A Smith KE Leventhal AM. Body dissatisfaction and binge eating: the moderating roles of sweet taste reward sensitivity and dietary restraint among tobacco product users. Int J Environ Res Public Health. (2022) 19:15523. doi: 10.3390/ijerph192315523, 36497598 PMC9740665

[24] Rolls ET. The orbitofrontal cortex, food reward, body weight and obesity. Soc Cogn Affect Neurosci. (2023) 18:nsab044. doi: 10.1093/scan/nsab044, 33830272 PMC9997078

[25] Fan H Wang P Zhang Y Huang G Nie Z Liu H . Effect of acute aerobic exercise on internet craving among college students with internet dependency: the mediating role of prefrontal cortex and executive control. Acta Psychol. (2025) 261:105912. doi: 10.1016/j.actpsy.2025.105912, 41252884

[26] Gellish RL Goslin BR Olson RE McDonald A Russi GD Moudgil VK. Longitudinal modeling of the relationship between age and maximal heart rate. Med Sci Sports Exerc. (2007) 39:822–9. doi: 10.1097/mss.0b013e31803349c6, 17468581

[27] Oustric P Thivel D Dalton M. Measuring food preference and reward: application and cross-cultural adaptation of the Leeds food preference questionnaire in human experimental research. Food Qual Prefer. (2020) 80:103824. doi: 10.1016/j.foodqual.2019.103824

[28] Killgore WD Killgore WDS Young AD Femia LA Bogorodzki P Rogowska J . Cortical and limbic activation during viewing of high- versus low-calorie foods. NeuroImage. (2003) 19:1381–94. doi: 10.1016/S1053-8119(03)00191-5, 12948696

[29] Strangman G Culver JP Thompson JH Boas DA New Collective Author. A quantitative comparison of simultaneous BOLD fMRI and NIRS recordings during functional brain activation. NeuroImage. (2002) 17:719–31. doi: 10.1006/nimg.2002.1227, 12377147

[30] Cheng C Yang Y. Food stimuli decrease activation in regions of the prefrontal cortex related to executive function: an fNIRS study. Eat Weight Disord. (2023) 28:96. doi: 10.1007/s40519-023-01623-7, 37982958 PMC10661783

[31] Domaradzka E Bielecki M. Deadly attraction - attentional bias toward preferred cigarette brand in smokers. Front Psychol. (2017) 8:1365. doi: 10.3389/fpsyg.2017.01365, 28848479 PMC5554524

[32] Zhang M Gao X Yang Z Han S Zhou B Niu X . Abnormal resting-state effective connectivity in reward network among long-term male smokers. Addict Biol. (2022) 27:e13221. doi: 10.1111/adb.13221, 36001421

[33] Yamada Y Hiratsu A Thivel D Beaulieu K Finlayson G Nagayama C . Reward for fat and sweet dimensions of food are altered by an acute bout of running in healthy young men. Appetite. (2024) 200:107562. doi: 10.1016/j.appet.2024.107562, 38880282

[34] Zheng L Miao M Gan Y. A systematic and meta-analytic review on the neural correlates of viewing high- and low-calorie foods among normal-weight adults. Neurosci Biobehav Rev. (2022) 138:104721. doi: 10.1016/j.neubiorev.2022.104721, 35667634

[35] Rolls ET Feng R Cheng W Feng J. Orbitofrontal cortex connectivity is associated with food reward and body weight in humans. Soc Cogn Affect Neurosci. (2023) 18:nsab083. doi: 10.1093/scan/nsab083, 34189586 PMC10498940

[36] Campos RC Marti F Rigoni D Fofo H Pousinha P Ortiz V . Nicotine disrupts top-down habenular control over cholinergic inputs to the ventral tegmental area to increase motivational valence of food rewards. Biol Psychiatry. (2026) 99:492–505. doi: 10.1016/j.biopsych.2025.06.036, 40659199

[37] Gunn MP Rose GM Whitton AE Pizzagalli DA Gilbert DG. Smoking progression and nicotine-enhanced reward sensitivity predicted by resting-state functional connectivity in salience and executive control networks. Nicotine Tob Res. (2024) 26:1305–12. doi: 10.1093/ntr/ntae084, 38624067 PMC11417123

[38] Dera AM Shen T Thackray AE Hinton EC King JA James L . The influence of physical activity on neural responses to visual food cues in humans: a systematic review of functional magnetic resonance imaging studies. Neurosci Biobehav Rev. (2023) 152:105247. doi: 10.1016/j.neubiorev.2023.105247, 37236384

[39] Thackray AE Hinton EC Alanazi TM Dera AM Fujihara K Hamilton-Shield JP . Exploring the acute effects of running on cerebral blood flow and food cue reactivity in healthy young men using functional magnetic resonance imaging. Hum Brain Mapp. (2023) 44:3815–32. doi: 10.1002/hbm.26314, 37145965 PMC10203797

[40] Cai Z Shi L Wu W Meng L Ru Y Wu M. A scoping review of effects of acute exercise on executive function: evidence from event-related potentials. Front Psychol. (2025) 16:1599861. doi: 10.3389/fpsyg.2025.1599861, 40376491 PMC12079670

[41] Ludyga S Schwarz A Leuenberger R Colombo S Kummer R Gerber M. Acute effects of aerobic exercise on risky decision making and reward processing in young adults. Psychophysiology. (2025) 62:e70029. doi: 10.1111/psyp.70029, 40028676

[42] Jung M Pontifex MB Hillman CH Kang M Voss MW Erickson KI . A mechanistic understanding of cognitive performance deficits concurrent with vigorous intensity exercise. Brain Cogn. (2024) 180:106208. doi: 10.1016/j.bandc.2024.106208, 39111187

[43] Jung M. Does prefrontal cortex oxygenation mediate executive function during high-intensity exercise? A systematic review of fNIRS studies. Behav Brain Res. (2025) 495:115765. doi: 10.1016/j.bbr.2025.115765, 40754149

[44] Yao S Lu H Zhang L Liu F Ma F Chi A. Acute exercise fatigue impairs cognitive control: neurophysiological mechanisms revealed by ERP and ERSP analyses. Biology. (2025) 14:1688. doi: 10.3390/biology14121688, 41463461 PMC12730307

[45] Li Y Sakazaki M Kamemoto K Nagayama C Miyashita M. The effect of different intensities of treadmill exercise on food reward in young men. Physiol Behav. (2025) 293:114844. doi: 10.1016/j.physbeh.2025.114844, 39952541

[46] Hsieh SS Bala A Layzell K Fatima Q Pushparajah C Maguire RK . Moderate-to-vigorous and light-intensity aerobic exercise yield similar effects on food reward, appetitive responses, and energy intake in physically inactive adults. Eur J Clin Nutr. (2025) 79:1204–10. doi: 10.1038/s41430-025-01574-5, 40021929 PMC12678172

[47] Tsai CL Chang YC Pan CY Wang TC Ukropec J Ukropcová B. Acute effects of different exercise intensities on executive function and oculomotor performance in middle-aged and older adults: moderate-intensity continuous exercise vs. high-intensity interval exercise. Front Aging Neurosci. (2021) 13:743479. doi: 10.3389/fnagi.2021.743479, 34720993 PMC8548419

